# Self-assembled, hemin-functionalized peptide nanotubes: an innovative strategy for detecting glutathione and glucose molecules with peroxidase-like activity

**DOI:** 10.1186/s40580-023-00356-8

**Published:** 2023-02-04

**Authors:** Song Xiang, Xincheng Long, Qiuxia Tu, Jian Feng, Xiaohe Zhang, Guangwei Feng, Li Lei

**Affiliations:** 1 Key Laboratory of Microbiology and Parasitology of Education, Department of Guizhou, School of Basic Medical Science, Guizhou Medical University, Guiyang, China; 2 School of Clinical Laboratory Science, Guizhou Medical University, Guiyang, 550025 China; 3 Department of Chemistry, Engineering Research Center for Molecular Medicine, School of Basic Medical Science, Guizhou Medical University, Guiyang, 550025 China; 4 School of Pediatrics, Guizhou Medical University, Guiyang, 550025 China

**Keywords:** Peptide nanotube, Hemin-functionalized, Peroxidase-like activity, Colorimetric, GSH and glucose

## Abstract

Accurately detecting dynamic changes in bioactive small molecules in real-time is very challenging. In this study, a hemin-based peptide assembly was rationally designed for the colorimetric detection of active small molecules. Hemin-functionalized peptide nanotubes were obtained through the direct incubation of hemin (hemin@PNTs) and peptide nanotubes (PNTs) or were coassembled with the heptapeptide Ac-KLVFFAL-NH_2_ via electrostatic, π–π stacking, and hydrophobic interactions (hemin-PNTs). This new substance is significant because it exhibits the benefits of both hemin and PNTs as well as some special qualities. First, hemin-PNTs exhibited higher intrinsic peroxidase-like activity, which, in the presence of H_2_O_2_, could catalyze the oxidation of the substrate 3,3',5,5'-tetramethylbenzidine (TMB) to yield a typical blue solution after 10 min at 25 ℃. Second, hemin-PNTs showed significantly higher activity than that of hemin, PNTs alone, or hemin@PNTs. Hemin-PNTs with a 20.0% hemin content may cooperate to improve catalytic activity. The catalytic activity was dependent on the reaction temperature, pH, reaction time, and H_2_O_2_ concentration. The nature of the TMB-catalyzed reaction may arise from the production of hydroxyl radicals. Fluorescence analysis was used to demonstrate the catalytic mechanism. According to this investigation, a new highly selective and sensitive colorimetric technique for detecting glutathione (GSH), L-cysteine, and glucose was established. The strategy demonstrated excellent sensitivity for GSH in the range of 1 to 30 μM with a 0.51 μM detection limit. Importantly, this glucose detection technique, which employs glucose oxidase and hemin-PNTs, is simple and inexpensive, with a 0.1 μM to 1.0 mM linear range and a 15.2 μM detection limit. Because of their low cost and high catalytic activity, hemin-PNTs are an excellent choice for biocatalysts in a diverse range of potential applications, including applications in clinical diagnostics, environmental chemistry, and biotechnology.

**Supplementary Information:**

The online version contains supplementary material available at 10.1186/s40580-023-00356-8.

## Introduction

In a living system, abnormal bioactive small molecules activity such as hydrogen peroxide (H_2_O_2_), biothiols and glucose can cause oxidative stress damage [1–8], which in turn can cause several diseases, including cancer, Parkinson's disease, and Alzheimer's disease [9–11]. Consequently, the ability to monitor the distribution and variations of bioactive small molecules in living systems in real-time can facilitate studies on disease mechanisms, which is extremely important for the early diagnosis and prevention of associated diseases [12]. Active small molecules, however, are unstable and highly reactive; thus, detecting these molecules in a complex environment is challenging. Consequently, developing and producing probes with high selectivity, high sensitivity, and quick response is a critical endeavor.

Artificial enzymes have been thoroughly studied and used in the development of biosensors over the last few decades [13–16]. In comparison to natural enzymes (such as horseradish peroxidase, HRP), nanomaterials offer many significant advantages, including high stability, ease of storage, low preparation costs, structure design, flexible composition, and tunable catalytic activities [17–23]. Nanomaterials are thus promising candidates for artificial peroxidase mimics. Therefore, artificial peroxidase mimetics with high catalytic activity have a promising future in enzyme-related fields. Additionally, it was discovered that some metal oxide nanomaterials, including copper-based nanoreactors [24], V_2_O_5_ nanowires [25], magnetic Fe_3_O_4_ nanoparticles [26], and CeO_2_ nanoparticles [27], exhibit intrinsic peroxidase-like activity. Moreover, carbon nanomaterials with intrinsic peroxidase catalytic activity have been found to include carbon nanotubes, carbon nanodots, graphene oxide, and multicolor luminescent carbon nanoparticles [28–32]. All these enzyme-like nanomaterials have been broadly used in a wide range of fields and techniques, such as immunoassays [33], biosensing [34], cancer diagnosis and treatment [35], neuroprotection [26], pollutant removal and stem cell growth [14, 36, 37]. However, although these enzyme mimics are certainly attractive, most are unstable and prone to aggregation in an aqueous solution. It is essential to search for stable, simple-to-prepare artificial enzymes that exhibit high catalytic activity.

Recent studies have focused much attention on peptide self-assembly materials that are based on the molecular self-assembly phenomenon [38]. Amphipathic biomimetic peptides can self-assemble into multifunctional nanostructures via π–π stacking and electrostatic and hydrophobic interactions [39]. The peptide assemblies display a wide variety of distinctive characteristics, including excellent biocompatibility, biodegradability, and the ability to exhibit tunable functionalities. Due to their periodic structure and uniform dimension, peptide nanotubes (PNTs) have been thoroughly investigated for a variety of applications, including light antennas, energy storage, catalysis, and drug delivery [40]. Previous studies have shown that, similar to the microenvironment of natural enzyme structures, polypeptide self-assembled nanostructures (fibers, nanotubes, etc.) can be used to simulate their catalytic active centers and to construct simulated enzymes with enzyme catalytic activity [41–43]. In addition, these nanostructures can be combined with some functional molecules, and their hydrophobic microenvironments can significantly enhance their catalytic activity [44, 45]. Amyloid assembly has recently been considered a possible scaffold for establishing enzyme analogs due to the advantageous properties of amyloids.

Hemin (iron protoporphyrin) is the active center of hemin proteins with peroxidase catalytic activity, including cytochromes peroxidase, hemoglobin, and myoglobin [46]. However, the catalytic activity and stability of hemin in water solubility are poor when compared with those of natural enzymes. To improve colorimetric detection, it is necessary to enhance the performance of hemin. Qiu and coworkers found that hemin/WS_2_ hybrid nanosheets could be prepared through π-π interactions with improved catalytic activity [47]. If hemin-functionalized peptide self-assembly materials could be obtained, it would improve the catalytic performance of hemin and peptide nanomaterials, thereby resolving the problem with hemin.

**Fig. 1:**
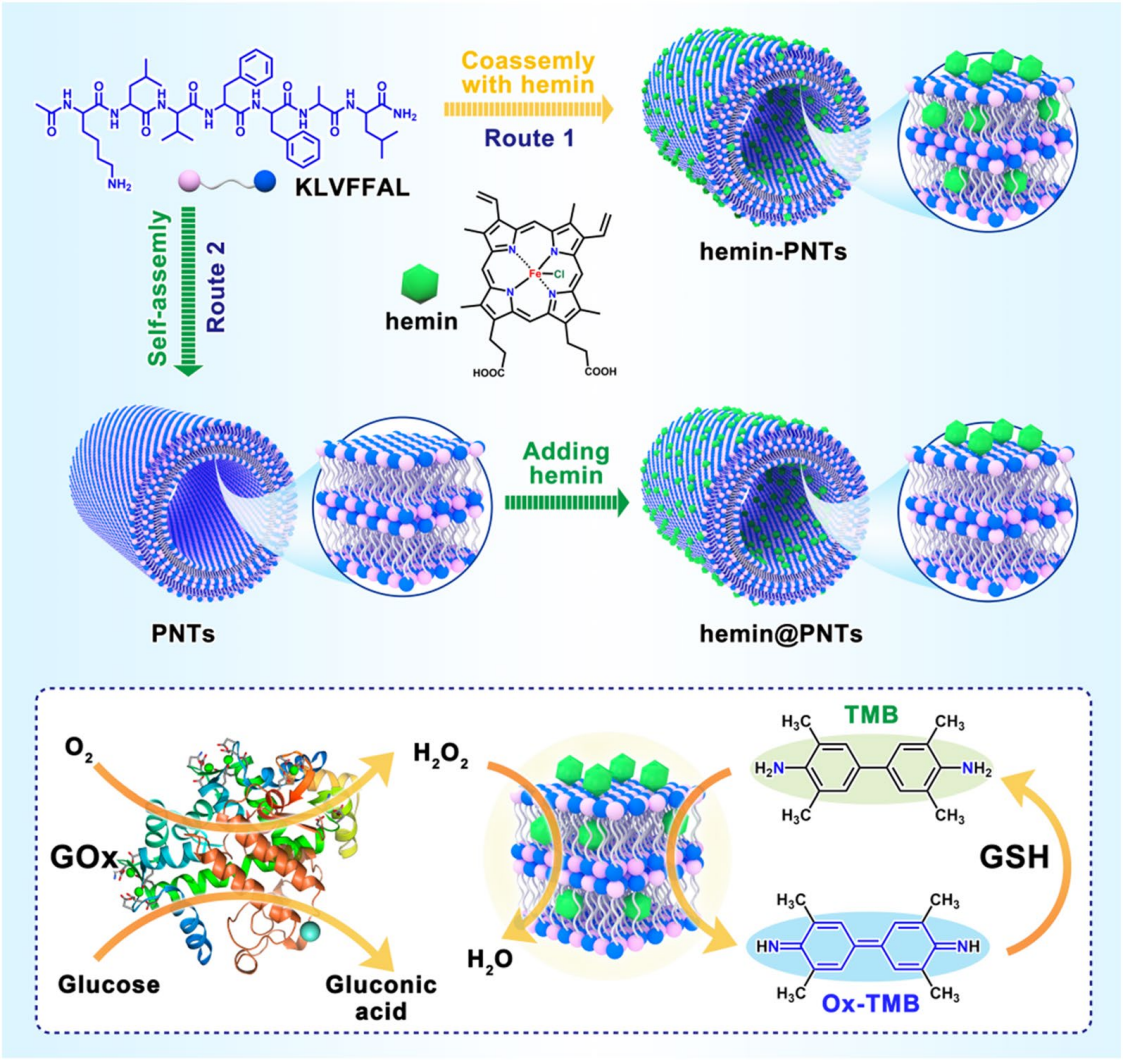
Schematic representation of hemin-functionalized peptide nanotubes and the design and principle of hemin-functionalized peptide nanotubes for the detection of GSH and glucose

In this study, the heptapeptide Ac-KLVFFAL-NH_2_ (KL-7) was utilized specifically for hemin loading. This peptide self-assembled at neutral pH into homogeneous PNTs [48]. Furthermore, the PNT out-of-register β-sheet structure exposed half of the surface lysine residues, creating a dense and well-organized amine array that could be further functionalized [41, 49, 50]. Hemin-functionalized PNTs were produced by incubating PNTs directly with hemin (produced hemin@PNTs) or by coassembling KL-7 with hemin (obtained hemin-PNTs). Significantly, hemin-functionalized PNTs possessed both excellent PNTs and hemin properties. The obtained PNTs also demonstrated increased catalytic activity. This excellent performance resulted from the ability of the PNTs scaffolds to prevent hemin dimerization, thereby enhancing its loading and activity. Based on these findings, the nanocomposite, as a novel peroxidase mimic, was employed to develop a simple, sensitive colorimetric approach for detecting glutathione (GSH), L-cysteine (Cys), and glucose in blood serum samples (Fig. 1).

## Materials and methods

### Chemicals and materials

The Ac-KLVFFAL-NH_2_ (KL-7) peptide was obtained from GL Biochem Ltd. (Shanghai). 3,3′5,5’-tetramethylbenzidine (TMB), 30% H_2_O_2_, anhydrous glucose, and hemin were acquired from Sinopharm Chemical Reagent Co, Ltd. (Shanghai, China). Reduced glutathione (GSH), glucose oxidase (GOx, 10 KU), L-glycine (Gly), L-cystine (Cys), L-alanine (Ala), L-isoleucine (Leu), L-valine, L-phenylalanine (Phe), L-arginine (Arg), L-threonine (Thr), fructose, galactose, sucrose, and maltose were obtained from Sigma Aldrich (Shanghai, China). Except when otherwise noted, all chemicals utilized were of analytical quality and required no further purification before use. The entire study was conducted using Milli-Q water.

### Instrumentation and characterization

Cary 60 Agilent Technologies was utilized to collect the UV‒vis absorption spectra. An Epoch2 microplate spectrophotometer (Bio Tek, Agilent, USA) was employed to obtain the kinetics curves. The fluorescence spectra were measured on an Agilent Technologies Cary Eclipse Fluorescence spectrophotometer (Santa Clara, CA, USA). Transmission electron microscopy (TEM) images of PNTs and hemin-functionalized PNTs were obtained using a Tecnai G2F30 Fourier transform Elmer spectrometer at an accelerating voltage of 200 kV. On an inverted Nikon Ti-U-DS-RI2 fluorescence microscope (Japan), fluorescence imaging was conducted. Circular dichroism (CD) spectra were obtained with a Chirascan spectropolarimeter (Applied Photophysics, UK).

### Synthesis of PNTs and hemin-functionalized PNTs

Assembly of KL-7 peptide (PNTs): The KL-7 peptide (3.5 mM) was ffrst dissolved in 1,1,1,3,3,3-hexaffuoro-2-propanol (HFIP) with continuous vortex and followed by sonication. After incubating in the ice bath for 30 min, the peptide films were dissolved in 333 μL of 4: 6 acetonitrile (ACN) /water (H_2_O) containing 0.1% triffuoroacetic acid and the pH was adjusted to 7.0 with 200 mM NaOH. Finally, it was allowed to assemble for 24 h in 37 °C water bath [48].

Hemin-functionalized PNTs: (i) For the coassembly of the KL-7 peptide with hemin (hemin-PNTs), hemin was first dissolved in dimethyl sulfoxide (DMSO) at the desired concentration (2.5, 5.0, 9.3, 14.9, and 18.6 mg/mL). Then, the hemin solution (10 μL of 2.5 mg/mL, 10 μL of 5.0 mg/mL, 10 μL of 9.3 mg/mL, 10 μL of 14.9 mg/mL, 10 μL or 13 μL of 18.6 mg/mL) was incorporated into 333 μL KL-7 peptide solutions (3.5 mM in ACN/H_2_O (40%) at pH 7.0), which were then incubated for 24 h at 37 °C water bath in the dark. (ii) For adsorption of hemin onto the surface of PNTs (hemin@PNTs), different concentrations of hemin (dissolved in DMSO) were incubated with 3.5 mM PNTs for 6 h at 37 °C water bath. Centrifugation (4 °C, 10,000 rpm) was then employed to wash the free hemin. UV‒vis spectroscopy was employed to ascertain the loading ratio and efficiency of hemin. The following equation was employed to calculate the LE [48]: LE (%) = (weight of loaded hemin)/(total weight of PNTs) × 100.

### Detection of H_2_O_2_ and glucose by hemin-functionalized PNTs as a mimicking enzyme

H_2_O_2_ was typically detected by adding hemin-PNTs (8 μL, 50 μg/mL), TMB (30 mM, 12 μL), and 60 μL Tris–HCl buffer (10 mM) at pH 5.0 to a 600 μL Eppendorf tube. Then, 40 μL of different concentrations of H_2_O_2_ were added. The mixed solutions were then incubated for 10 min at room temperature. The amount of H_2_O_2_ was then measured using the mixture's absorbance at 652 nm. Each experiment was performed three times, and five measurements of each sample solution were obtained.

The following procedure was used to detect glucose: 6 μL of GOx solution (10 mg/mL) was placed into an Eppendorf tube (600 μL), followed by 44 μL of a solution containing different concentrations of glucose in 10 mM Tris–HCl buffer (pH 7.0). This mixture was then maintained at 37 °C for 30 min to yield H_2_O_2_. Then, TMB (12 μL, 30 mM), 50 μL of Tris–HCl buffer (10 mM, pH 5.0), and hemin-PNTs (8 μL, 50 μg/mL) were successively poured into the resulting solution, and the mixture was further incubated for 10 min at room temperature. Finally, the absorbance was measured at 652 nm. In the control experiment, fructose, galactose, sucrose, and maltose were used as test interferences.

### Detection of GSH and Cys

GSH was detected as follows: 8 μL of 50 μg/mL hemin-PNTs, 40 μL of GSH at various concentrations, 40 μL of H_2_O_2_ (18 mM), 12 μL of TMB (3 mM) and 20 μL of Tris–HCl buffer (pH 5.0, 10 mM) were reacted for 10 min at room temperature prior to obtaining spectroscopy measurements. Cys detection was comparable to GSH detection. K^+^, Na^+^, Gly, Ala, Val, Leu, Phe, and Arg were used as test interferences in control experiments.

### Detection of glucose in a human serum sample

Samples of serum from healthy humans were used to detect glucose in complex biological samples using both the experimental method described above and the standard addition approach; the reaction solution contained 12 μL of serum samples, 12 μL of GOx, and 36 μL of Tris–HCl buffer (pH 7.0, 10 mM). The mixed solution was maintained at 37 °C for 30 min. Subsequently, 24 μL of Tris–HCl buffer (10 mM), 8 μL of 50 μg/mL hemin-PNTs, 12 μL of TMB (3 mM), and 16 μL of Tris–HCl buffer (pH 7.0) were added to the aforementioned solution and incubated for 30 min at 37 °C. UV‒visible absorption spectra were used to monitor the mixture's absorbance at 652 nm.

## Results and discussion

### Characterization of KL-7 nanotubes

**Fig. 2:**
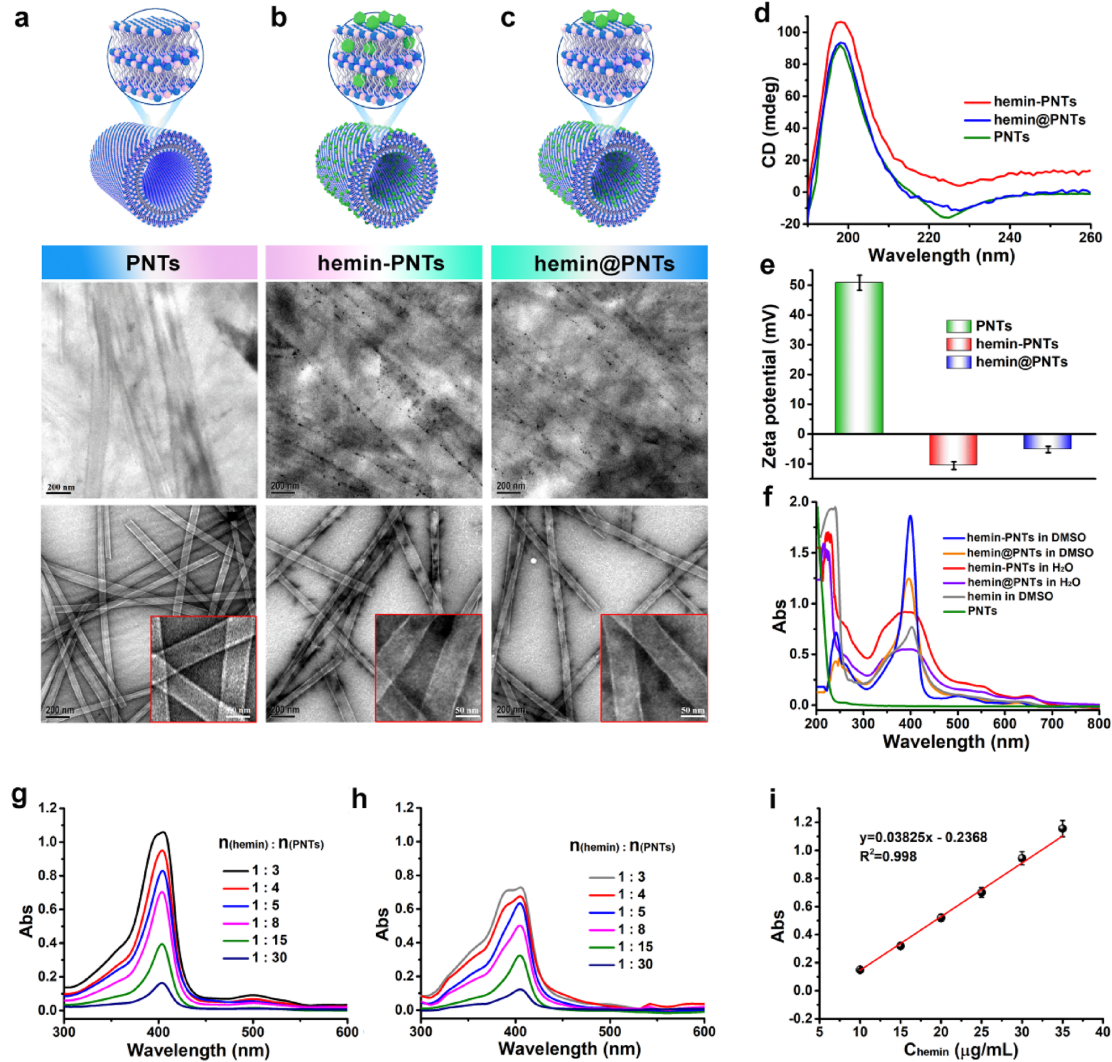
Images from the top to the bottom for structure and TEM imaging with or without negative staining: there are **a** PNTs, **b** hemin-PNTs, and **c** hemin@PNTs. **d** CD spectra of PNTs, hemin-PNTs, and hemin@PNTs. **e** Zeta potential of PNTs, hemin-PNTs, and hemin@PNTs. **f** UV‒vis spectra of the PNTs, hemin-PNTs, and hemin@PNTs. UV‒vis spectra of **g** hemin-PNTs and **h** hemin@PNTs with various hemin:PNT molar ratios. **i** Standard curve of hemin. Data represent means ± SDs. (n = 3)

The PNTs were produced using a method described previously [45]. In brief, KL-7 monomers containing the peptide sequence Ac-KLVFFAL-NH_2_ (Fig. 1) were self-assembled in a solution of ACN/H_2_O at neutral pH (pH 7.0), and 24 h of room temperature incubation resulted in the formation of mature nanotubes. Using TEM, SEM and Nile red staining (Fig. 2a, Additional file 1: Figs .S1, S2a), the formation of PNTs with a homogeneous tubular morphology with an average length of several micrometers and a diameter of 50 nm was observed. It was confirmed by CD spectra in Fig. 2d that antiparallel and out-of-register β-sheets had formed, which greatly increased the potential of PNTs for entrapping small molecules.

### Characterization of hemin-functionalized PNTs

On the surface of PNTs, half of the lysine residues were exposed due to their out-of-register β-sheet structure, which creates a dense and organized amine array for hemin loading. Hemin was incorporated into PNTs either before or after the KL-7 monomer was assembled to create hemin-functionalized peptide nanotubes, which were designated hemin-PNTs or hemin@PNTs, respectively. Initially, TEM and SEM images were used to examine the capacity of hemin-loaded PNTs to form nanostructures. The SEM images of the as-prepared PNTs, hemin-PNTs and hemin@PNTs were shown in Fig. S1, which were used to confirm the nanotube structure of hemin-functionalized PNTs, indicating that hemin molecules loaded in PNTs would not break their original nanotube morphology. The TEM images following negative staining further demonstrated that the hemin-functionalized PNTs and PNTs exhibit comparable tube morphology, as shown in Fig. 2b and c. The surface charge of these nanotubes was further characterized using zeta potential measurements. The change from positively charged PNTs (50.8 ± 0.5 mV) to negatively charged hemin-PNTs (− 10.6 ± 0.2 mV) and hemin@PNTs (− 5.2 ± 0.6 mV) suggested that hemin was loaded onto the peptide nanotube, as shown in Fig. 2e. Both the hemin-PNTs and the hemin@PNTs showed evidence of a β-sheet conformation, according to CD (Fig. 2d), indicating that the secondary structure conformation of the PNTs was unaffected by the functionalization of hemin.

To prepare hemin-functionalized PNTs, a variety of hemin-to-PNT molar ratios were evaluated. After modification, the dispersion of PNTs changed from transparent to brown (Additional file 1: Fig. S2b). The presence of the hemin absorption peak in hemin-PNTs and hemin@PNTs indicated that it was successfully loaded into the PNTs (Fig. 2f and Additional file 1: Fig. S2) [51]. The UV‒vis absorption spectra shown in Fig. 2g,h and 2i were used to calculate the hemin loading efficiencies (LEs, Additional file 1: Table S1), which demonstrated that the LEs and feeding ratio of hemin to PNTs were positively correlated and indicated that LEs in the range of 2.3–20.0% were possible. However, the maximum absorption from hemin@PNTs (Fig. 2h) was always lower than that from hemin-PNTs (Fig. 2g), indicating that less hemin was present on the PNTs surface. The LEs in Additional file 1: Table S1 also revealed that the hemin loading of hemin-PNTs produced by assembling was greater than that of hemin@PNTs produced by simple adsorption.

### Peroxidase-like activity evaluation

**Fig. 3:**
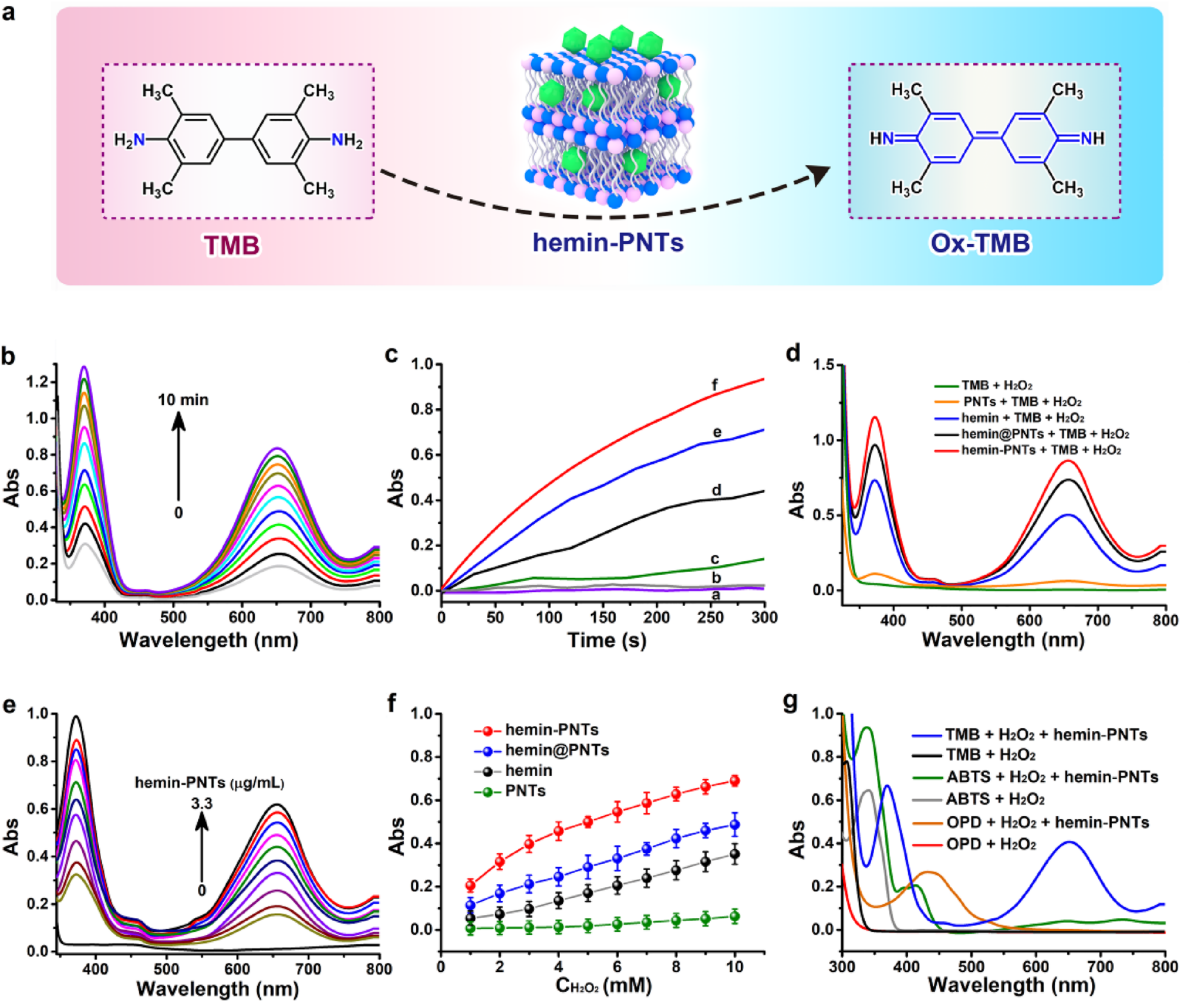
**a** Schematic depiction of hemin-PNTs as a nanoenzyme. **b** The UV‒vis absorption spectra of hemin-PNTs + TMB + H_2_O_2_ change as the reaction time increases. The concentrations of hemin-PNTs, TMB, and H_2_O_2_ were 3.3 μg/mL, 3 mM, and 10 mM, respectively. **c** Changes in the absorbance of TMB solution over time at 652 nm in the following reaction systems: a. TMB + H_2_O_2_; b. hemin-PNTs + TMB; c. PNTs + TMB + H_2_O_2_; d. hemin + TMB + H_2_O_2_; e. hemin@PNTs + TMB + H_2_O_2_; and f. hemin-PNTs + TMB + H_2_O_2_. **d** Absorption spectra of TMB–H_2_O_2_ mixed solution in the absence of a catalyst (black line) and in the presence of 3.3 μg/mL PNTs (green line), 3.3 μg/mL hemin (blue line), 3.3 μg/mL hemin@PNTs (green line) and 3.3 μg/mL hemin-PNTs (red line). **e** Changes in the absorbance of a 10 mM H_2_O_2_ and 3 mM TMB solution are shown at different hemin-PNT concentrations. **f** The catalytic activity of hemin, PNTs, hemin@PNTs, and hemin-PNTs at various concentrations of H_2_O_2_. **g** TMB, OPD, and ABTS absorption spectra changed as a result of hemin-PNT-catalyzed oxidation. Data represent means ± SDs (n = 3)

Peroxidase, which is commonly known, can catalyze peroxidase substrate oxidation, resulting in a color change (Fig. 3a) [52]. Hemin-functionalized PNTs activity similar to that of peroxidase was evaluated using the TMB-H_2_O_2_ reaction as a model reaction system. Initially, the peroxidase-like activity of the hemin-functionalized PNTs was measured by their ability to catalyze the oxidation of TMB peroxidase substrates in the presence of H_2_O_2_. This reaction yields the characteristic blue color of peroxidase reactions and deepens with reaction time (Fig. 3b, c, and Additional file 1: Fig. S3a), reaching its maximum absorbance at 369 nm and 652 nm, respectively. However, no oxidation reaction was observed when H_2_O_2_ or PNTs were added to TMB solution with 652 nm absorbance (Fig. 3c and d). These findings provided strong evidence that hemin-functionalized PNTs displayed intrinsic peroxidase-like activity and that, like HRP, they need both H_2_O_2_ and hemin-functionalized PNTs to catalyze the reaction.

Hemin alone or PNTs functionalized with hemin alone were also tested to compare how the TMB-H_2_O_2_ system responded. Hemin is the active site of peroxidase enzymes, and it exhibits catalytic properties that are analogous to those of peroxidase. As seen in Fig. 3c and d, it was also found that PNTs exhibited intrinsic peroxidase-like activity. In addition, Fig. 3c and d illustrates a comparative study in which the TMB–H_2_O_2_ system's response was examined in the presence of hemin alone and PNTs alone. According to the findings, the hemin-functionalized PNTs were significantly more effective than either hemin or PNTs alone. Significantly, when hemin, hemin/PNTs, and hemin-PNTs were added at the same concentration gradient, the absorption of TMB at 652 nm gradually decreased in the order hemin-PNTs > hemin/PNTs > hemin, indicating that hemin-PNTs exhibited the strongest catalytic activity. The TMB–H_2_O_2_ reaction gradually accelerated as the hemin-PNT concentration increased from 0 to 3.3 μg/mL (Fig. 3e and Additional file 1: Fig. S4a). The catalytic activity of hemin-PNTs was further investigated by comparing the activity to that of hemin, PNTs, and hemin@PNTs (at the same amount of hemin) at various H_2_O_2_ concentrations (Fig. 3f). It was discovered that the activity of the hemin-PNTs was significantly higher than that of hemin, PNTs, or hemin@PNTs. Hemin-functionalized PNTs therefore exhibit high catalytic activity due to the synergistic effects between hemin and PNTs. All subsequent experiments were conducted with hemin-PNTs.

The catalytic oxidation of additional peroxidase substrates, such as OPD and ABTS, in the presence of H_2_O_2_ served as additional evidence of the peroxidase-like activity of hemin-PNTs. Figure 3g displays the outcomes. Hemin-PNTs could catalyze ABTS to produce a green color and OPD to produce an orange color, as shown in Fig. S5.

**Fig. 4:**
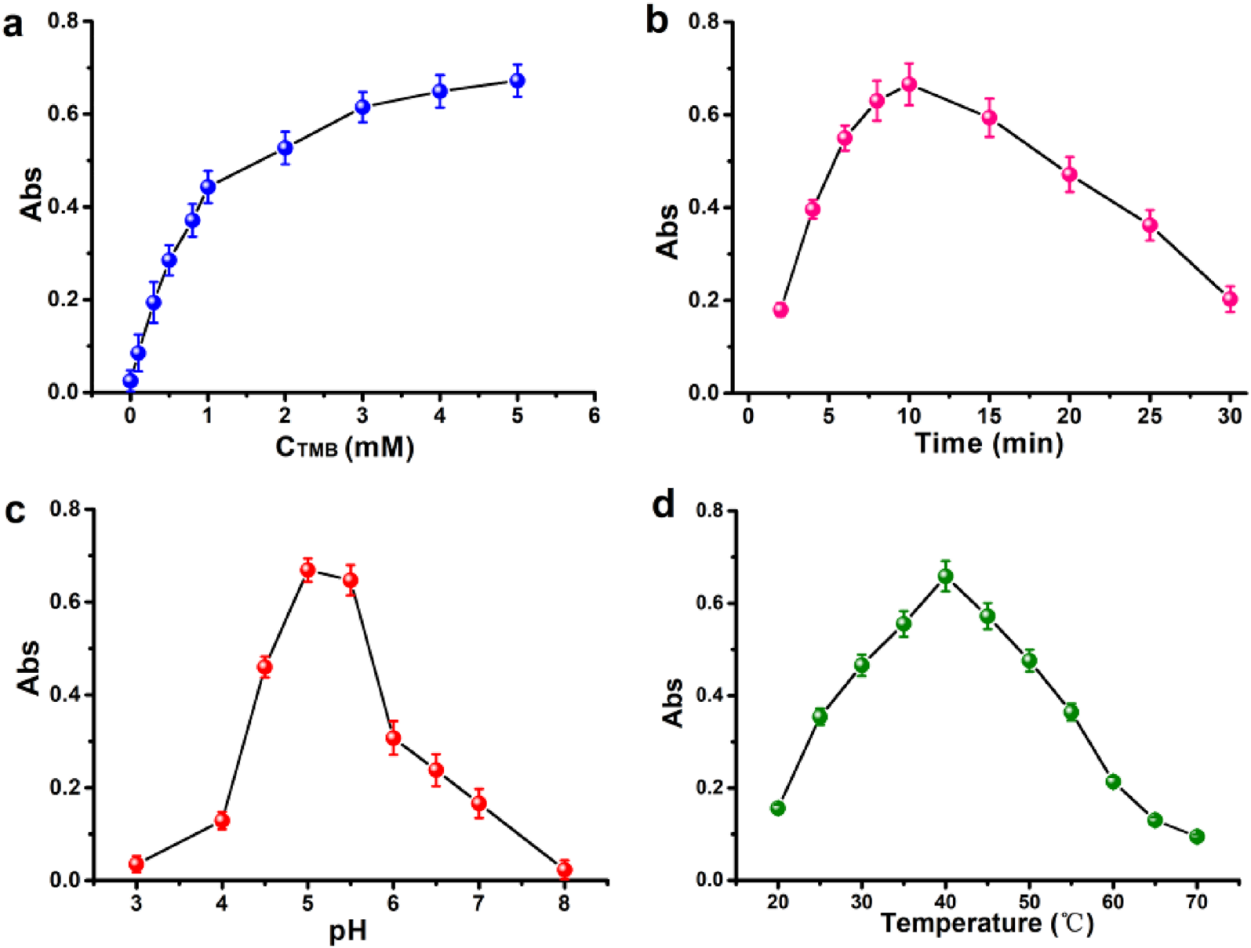
Impact of the TMB reaction (**A**), time (**B**), pH (**C**), and temperature (**D**) on the peroxidase-like activity of hemin-PNTs for TMB oxidation. The experiment was conducted with 3.3 μg/mL hemin-PNTs in 1.0 mL Tris–HCl buffer (1 mM, pH 5.0) containing 3 mM TMB and 6 mM H_2_O_2_ for 10 min at 40 °C. Data represent means ± SDs (n = 3)

As a proof-of-concept reaction system, the substrates H_2_O_2_ and TMB were employed to further enhance the activity of hemin-PNTs that resemble peroxidase. The concentration of TMB was initially optimized at 3 mM (Fig. 4a and Additional file 1: Fig. S6a). Hemin-PNTs catalytic activity was, as anticipated, influenced by pH and temperature, similar to HRP and NP-based peroxidase mimics. A greater catalyst concentration and longer reaction time resulted in greater catalytic activity. After initially increasing with reaction time, the catalytic activity slowed after 10 min (Fig. 4b and Additional file 1: Fig. S6b). According to reports, after incubation at pH levels less than 4.0 or temperatures greater than 50 °C, HRP's catalytic activity was significantly reduced [53]. In contrast, hemin-PNTs demonstrated significant catalytic activity over a wide pH (3.0–8.0) and temperature (20–65 °C) range, and they may be useful in harsh environments due to their robust tubular structure (Fig. 4c, d and Additional file 1: Fig. S6c, d). Further, to obtain the correlation between loading efficiency and the activity, the catalytic activity of hemin-PNTs with various hemin content was performed by TMB-H_2_O_2_ reaction system. As shown in Additional file 1: Fig. S8, the catalytic activity of hemin-PNTs increased with the increase of hemin loading efficiency.The optimal concentration of TMB, reaction time, temperature, pH and loading efficiency for this investigation's experimental conditions were 3 mM, 10 min, 40 °C, 5.0 and 20.0%, respectively.

It has been thoroughly demonstrated that in the presence of^**.**^OH, nonfluorescent terephthalic acid (TA) is transformed to 2-hydroxylterephthalic acid (HTA), a highly fluorescent molecule [54, 55]. Then, 3.3 μg/mL hemin-PNTs were added to 1 mM, 2 mM, and 3 mM H_2_O_2_ for a one-hour reaction at room temperature. After being centrifuged at 12,000 rpm for 30 min at 4 °C, the supernatant was collected, and a fluorescence spectrum at an excitation wavelength of 320 nm was obtained. As depicted in Additional file 1: Fig. S9, TA in 40 mM PBS buffer (pH 7.4) did not produce a characteristic peak, and a characteristic peak in the presence of H_2_O_2_ was also not observed. In the presence of hemin-PNTs in different H_2_O_2_ concentrations, however, a dramatic increase in the fluorescence signal at approximately 425 nm was observed in the fluorescence spectra in Fig. S9, indicating that hemin-PNTs could efficiently promote H_2_O_2_ to produce^**.**^OH, causing the TMB oxidation reaction.

### Detection of H_2_O_2_ and glucose

**Fig. 5:**
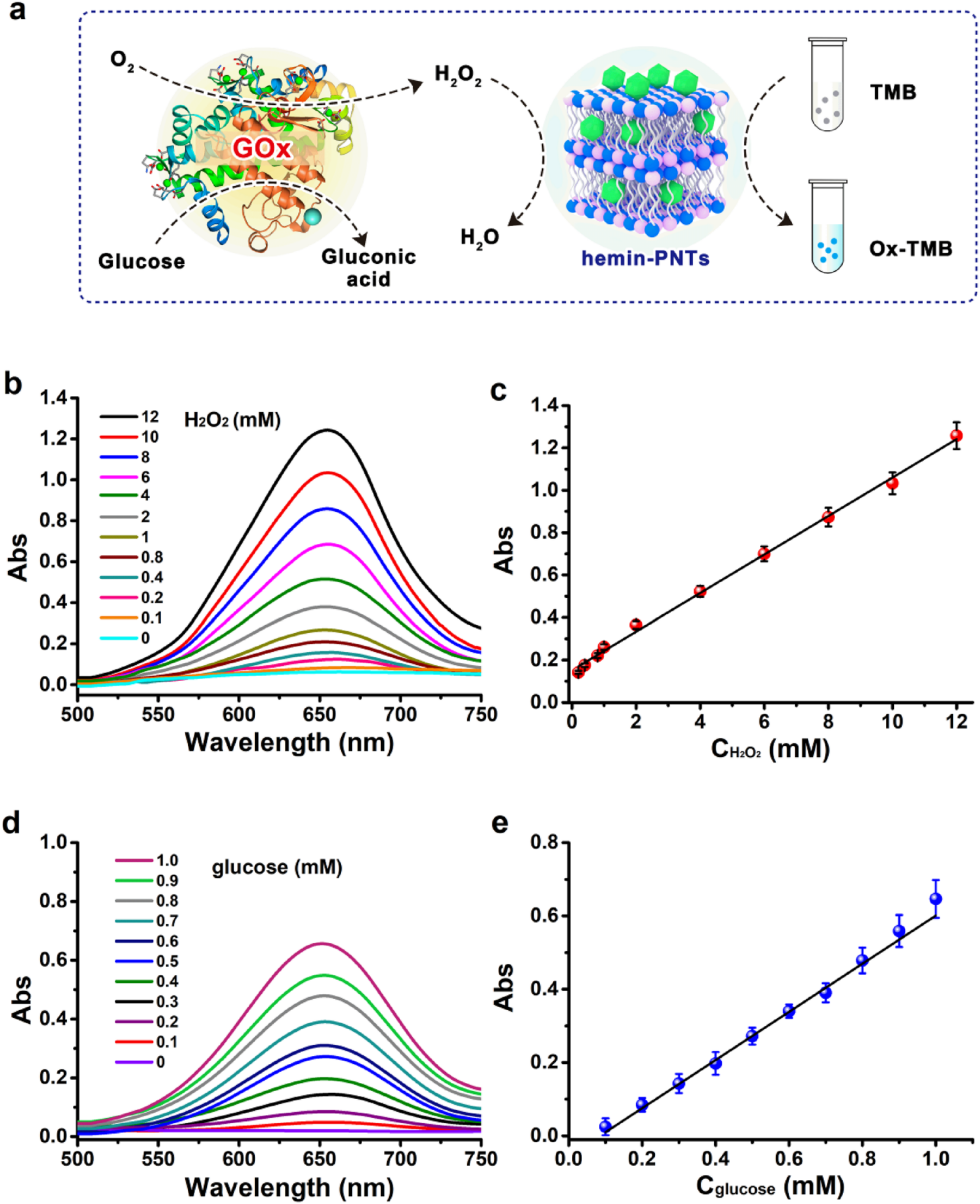
**a** Schematic representation of colorimetric glucose detection using glucose oxidase (GOx) and hemin-PNTs-catalyzed reactions. The UV‒vis spectrum of hemin-PNTs (3.3 μg/mL) at various concentrations of **b** H_2_O_2_ and **d** glucose with a constant amount of TMB (3 mM). The linear calibration curve for **c** H_2_O_2_ and **e** glucose. Data represent means ± SDs. (n = 3)

The sensitivity of the suggested label-free colorimetric technique for detecting H_2_O_2_ and glucose was assessed based on the aforementioned optimum assay conditions (Fig. 5a). After various H_2_O_2_ concentrations were examined, a typical H_2_O_2_ concentration-dependent curve for the change in absorbance was obtained, as shown in Fig. 5b, at 652 nm. The absorbance values increased as the H_2_O_2_ concentration increased. From 0.1 to 12 mM, a strong linear correlation between the H_2_O_2_ concentration and the absorption intensity was observed, as shown in Fig. 5c. Three times the standard deviation correlating to the detection of blank samples yielded an estimate of a detection limit of 1.3 μM.

When used in conjunction with GOx, GOx catalyzed glucose oxidation to gluconic acid (Fig. 5a) [56]. Additionally, oxygen in the solution was converted into H_2_O_2_, which then catalyzed the oxidation of TMB to develop a blue-colored product using hemin-functionalized peptide nanotubes. This indicated that the suggested label colorimetric method could also be applied to measure glucose, a crucial marker for the clinical diagnosis of diabetes [57]. Various glucose concentrations between 0.1 mM and 1.0 mM were examined using UV‒vis spectra, as shown in Fig. 5d. With increasing glucose concentrations, the solution's absorption intensity at 652 nm increased gradually. As the limit of detection was 15.2 μM and the linear range for glucose was 0.1 to 1.0 mM (Fig. 5d), a more sensitive measurement was obtained than those reported in most previous reports. A comparison of the absorption intensities introduced on by lactose, fructose, and maltose was carried out to further investigate the selectivity of this new glucose sensing system. The findings showed that even at 5 mM concentrations, these glucose analogs produced a negligible absorbance when compared to that of glucose, as shown in Fig. S10. This demonstrated the high selectivity of this sensing system for glucose detection, which may be related to glucose oxidase's high affinity for glucose.

Enzyme stability is a key problem that needs attention. Cyclic experiment in here was conducted to investigate re-usability of hemin-PNTs. The results showed that the catalytic activity of hemin-PNTs was above 90% after 10 cycles (Additional file 1: Fig. S11). Hence, hemin-PNTs exhibited conspicuous catalytic activity and stability could be a hopeful alternative for natural enzymes.

### Detection of GSH and Cys

**Fig. 6:**
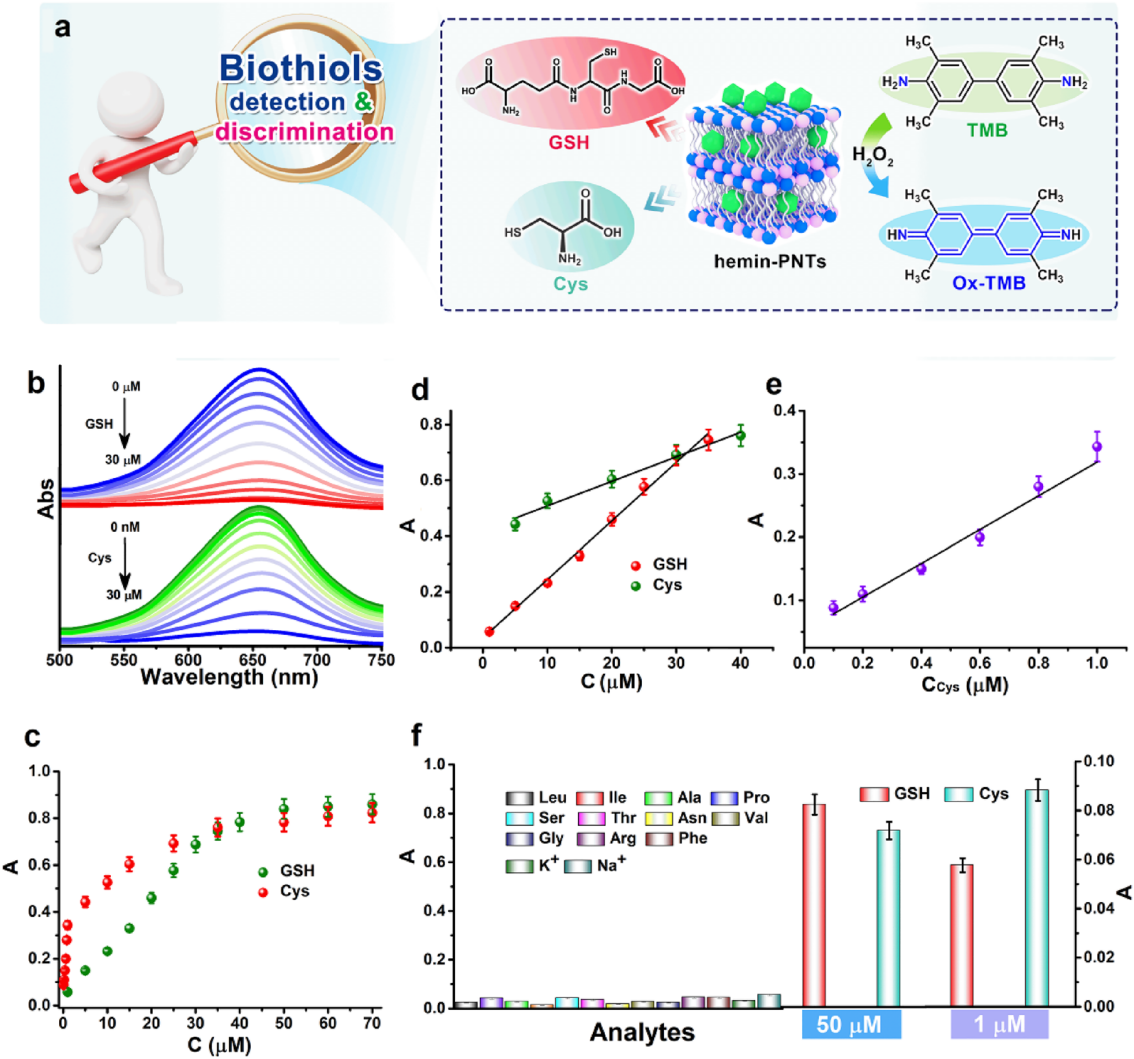
**a** Schematic representation of colorimetric GSH and Cys detection or discrimination using hemin-PNT-catalyzed reactions. **b** UV‒vis spectra of TMB + H_2_O_2_ + hemin-PNTs in the presence of various concentrations of GSH and Cys (0–30 μM). **c** A dose‒response curve for the detection of GSH and Cys with hemin-PNTs. **d** The linear response of GSH and Cys. **e** The linear response of Cys over the concentration range of 0.1 μM to 1 μM. **f** Monitoring the absorbance at 652 nm allows for the selective detection of GSH and Cys. The value of A is defined as A_0_ − A_I_, and A_0_ and A_I_ represent the TMB + H_2_O_2_ + hemin-PNT system with or without GSH and Cys, respectively. Data represent means ± SDs. (n = 3)

As a thiol molecule with antioxidant activity, GSH can directly reduce H_2_O_2_ to H_2_O, resulting in conversion of the colored oxidized substrate (ox-TMB) to the colorless reduced substrate (TMB) through its own antioxidation [58]. In addition, the active sites of hemin can be inhibited by the absorption of GSH's oxidation product. This inhibits the peroxidase-like activity of hemin-PNTs. Cys and GSH share comparable properties [59]. The peroxidase-like activity of hemin-PNTs was then utilized for the quantitative colorimetric detection and discrimination of GSH and Cys (Fig. 6a). As the concentration of GSH increased (Fig. 6b), A_652_ gradually declined. The value of *A* = *A*_*0*_–*A*_*I*_ demonstrated a linear relationship with the concentration of GSH between 1 and 35 μM (Fig. 6b and c). The GSH detection limit was 0.51 μM. This method was also employed to detect Cys. The UV‒vis spectra of 10 nM to 30 μM Cys are displayed in Fig. 6b, c. Figure 6e depicts typical concentration‒response curves for Cys with linear ranges of 100 nM–1 μM (Fig. 6e), 5–40 μM (Fig. 6d) and detection limits of 62 nM and 891 nM, respectively, which are sensitive for Cys detection. For the selectivity test, several common amino acids and interference ions were measured. The concentration of the interfering substance was 10 times greater than the concentration of the test substance (Fig. 6f). This method was selective toward GSH and Cys. However, when the concentrations of Cys and GSH reached 50 μM, the responses of all these methods were significantly increased (Fig. 6f)). When the concentration of Cys was decreased to 1 μM (Fig. 6f), the Cys response increased, achieving selective detection of Cys and GSH in the range of 200 nM–1 μM.

### Application of hemin-PNTs in serum samples

**Fig. 7:**
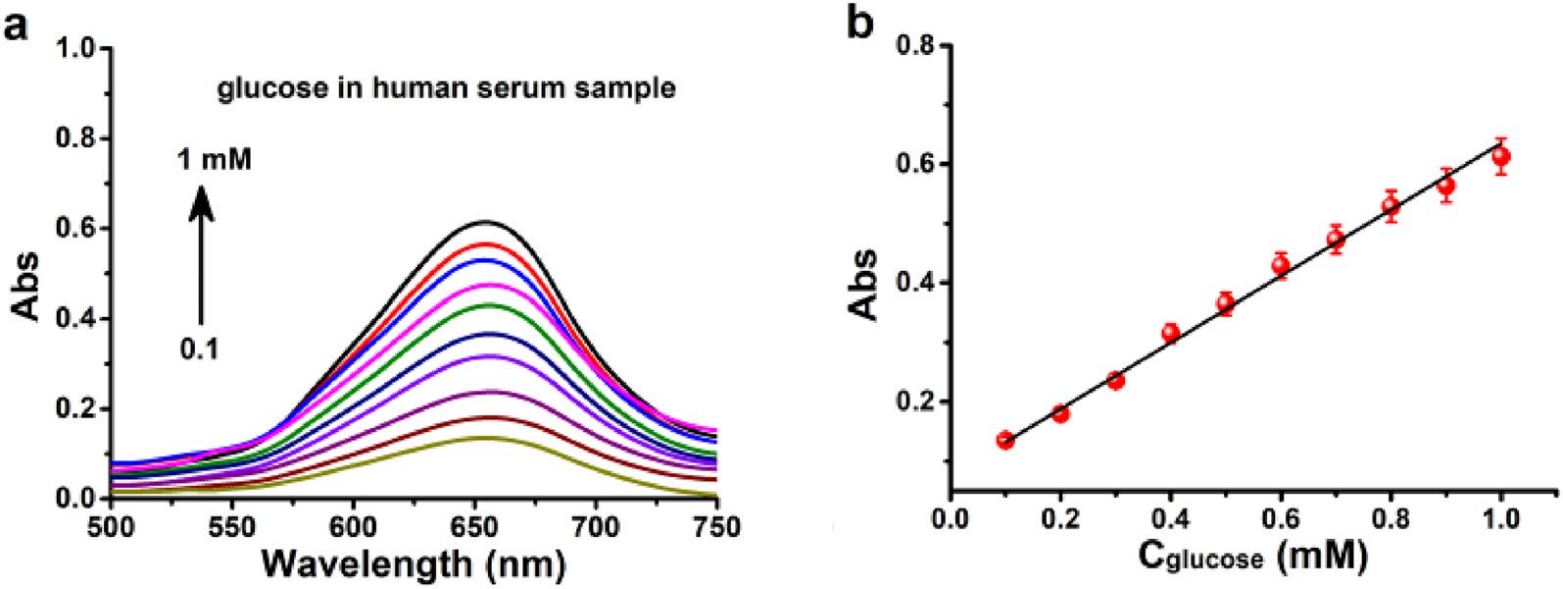
**a** UV‒vis spectra of different concentrations of glucose in human serum samples (0.1–1 mM). **b** Calibration curve for glucose, which shows a linear relationship. Data represent means ± SDs. (n = 3)

The present label-free colorimetric assay was employed to measure the amount of glucose in three samples of healthy individuals’ serum; this was performed to assess the applicability of the suggested technique based on the hemin-PNTs to measure glucose in complex biological samples [60]. Figure 7 and Table 1 display the outcomes of the determination and recovery. The relative standard deviations were between 1.2% and 5.1%, and the recoveries ranged from 95.0% to 105%. These outcomes demonstrated the validity of the suggested label-free colorimetric method to quantify glucose in real biological samples.

**Table 1:** The findings of the analysis of glucose concentration in human serum samples

Sample number	Added (mM)	Detection (mM)	Recovery (%)	RSD (%, n = 5)
1	0.2	0.21	105	5.1
2	0.4	0.36	103	1.2
3	0.6	0.57	95	1.5

## Conclusion

In conclusion, it was reported for the first time that hemin-functionalized peptide nanotubes exhibit intrinsic peroxidase-like activity, and these nanotubes are characterized by excellent stability, rapid response, and potent catalytic activity. Furthermore, KL-7 nanotubes were introduced as a new hemin support, and their various affinities when interacting with hydrophobic-like molecules were demonstrated. In comparison to HRP and other peroxidase nanomimetics, the hemin-PNTs showed some advantages, including ease of preparation, dispersibility, stability, low cost, and high catalytic efficiency. The hemin-PNTs were demonstrated to be both sensitive and selective in the detection of GSH, Cys, and glucose, which occurred via colorimetric methods without a label. Considering these benefits, it is believed that the newly synthesized hemin-PNTs could be utilized in biotechnology, environmental chemistry, and clinical diagnostics.

## Supplementary Information

**Additional file 1: Fig. S1**. SEM images of PNTs, hemin-PNTs and hemin@PNTs, scale bars = 250 nm. **Fig. S2.** (a) Fluorescence micrograph of 1 mM PNTs incubated with 10 µM Nile red. (b) Photographs of PNTs, hemin, hemin-PNTs and hemin@PNTs. **Fig. S3.** UV–vis absorbance spectra of hemin (0-40 µg/mL). **Fig. S4.** (a) The color change in the system of hemin-PNTs + TMB + H2O2 with the reaction time. (b) The optical photographs of the TMB–H2O2 mixed solution in the absence of a catalyst (1), and in the presence of PNTs (2), hemin (3), hemin@PNTs (4) and hemin-PNTs (5), respectively. **Fig. S5.** The optical photographs of the TMB–H2O2 mixed solution in the presence of increased concentration of hemin-PNTs (a) and hemin@PNTs (b). **Fig. S6.** Hemin-PNTs-catalyzed oxidation of various substrates showing changes in color: (a) TMB, (b) OPD, (c) ABTS. **Fig. S7.** Effect of reaction TMB (a), time (b), pH(c), and temperature (d) on the peroxidase-like activity of hemin-PNTs for the TMB oxidation. The experiment was carried out using 3.3 μg/mL hemin-PNTs in a reaction volume of 1.0 mL, in tris-HCl buffer (1 mM, pH 5.0) with 3.0 mM TMB and 1 mM H2O2 for 10 min at 40 °C. **Fig. S8.** The catalytic activity of hemin-PNTs with different hemin loading efficiency. **Fig. S9.** A Proof for the enhanced generation of .OH radicals by photolutuminesence spectral changes of terephthalic acid solution in the presence of H2O2 with different concentration. **Fig. S10.** A Selectivity analysis glucose, detection by monitoring the absorbance at 652 nm. The analyte concentrations were as follows: 1 mM glucose, 10 mM the concentration of interferences ( sucrose, fructose, maltose, galactose). **Fig. S11.** Recyclability of hemin-PNTs. **Table S1.** The loading efficiencies (LE %) calculated by formula of LE (%) = (weight of loaded hemin)/(total weight of PNT) × 100%.

## Data Availability

The authors have no data to share since all data are shown in the submitted manuscript.
