# Supplementary material for: Self-assembled, hemin-functionalized peptide nanotubes: an innovative strategy for detecting glutathione and glucose molecules with peroxidase-like activity
Source: Nano Converg. 2023 Feb 4;10:7. doi: 10.1186/s40580-023-00356-8 (PMC9899300; doi:10.1186/s40580-023-00356-8)
Supplement: Supplementary file 1 — Additional file 1: Fig. S1. SEM images of PNTs, hemin-PNTs and hemin@PNTs, scale bars = 250 nm. Fig. S2. (a) Fluorescence micrograph of 1 mM PNTs incubated with 10 µM Nile red. (b) Photographs of PNTs, hemin, hemin-PNTs and hemin@PNTs. Fig. S3. UV–vis absorbance spectra of hemin (0-40 µg/mL). Fig. S4. (a) The color change in the system of hemin-PNTs + TMB + H2O2 with the reaction time. (b) The optical photographs of the TMB–H2O2 mixed solution in the absence of a catalyst (1), and in the presence of PNTs (2), hemin (3), hemin@PNTs (4) and hemin-PNTs (5), respectively. Fig. S5. The optical photographs of the TMB–H2O2 mixed solution in the presence of increased concentration of hemin-PNTs (a) and hemin@PNTs (b). Fig. S6. Hemin-PNTs-catalyzed oxidation of various substrates showing changes in color: (a) TMB, (b) OPD, (c) ABTS. Fig. S7. Effect of reaction TMB (a), time (b), pH(c), and temperature (d) on the peroxidase-like activity of hemin-PNTs for the TMB oxidation. The experiment was carried out using 3.3 μg/mL hemin-PNTs in a reaction volume of 1.0 mL, in tris-HCl buffer (1 mM, pH 5.0) with 3.0 mM TMB and 1 mM H2O2 for 10 min at 40 °C. Fig. S8. The catalytic activity of hemin-PNTs with different hemin loading efficiency. Fig. S9. A Proof for the enhanced generation of .OH radicals by photolutuminesence spectral changes of terephthalic acid solution in the presence of H2O2 with different concentration. Fig. S10. A Selectivity analysis glucose, detection by monitoring the absorbance at 652 nm. The analyte concentrations were as follows: 1 mM glucose, 10 mM the concentration of interferences ( sucrose, fructose, maltose, galactose). Fig. S11. Recyclability of hemin-PNTs. Table S1. The loading efficiencies (LE %) calculated by formula of LE (%) = (weight of loaded hemin)/(total weight of PNT) × 100%. [file 40580_2023_356_MOESM1_ESM.docx]

**Supplementary material**

**Self-assembled, Hemin-functionalized peptide nanotubes: an innovative strategy for the detection of glutathione and glucose with peroxidase-like activity**

Song Xiang^1, §^, Xincheng Long^2, §^, Qiuxia Tu^3^, Jian Feng^3^, Xiaohe Zhang^4^, Guangwei Feng^3^, Li Lei^3^

^1^ Key Laboratory of Microbiology and Parasitology of Education Department of Guizhou, School of Basic Medical Science, Guizhou Medical University

^2^ School of Clinical Laboratory Science, Guizhou Medical University, Guiyang 550025, China

^3^ Department of Chemistry, Engineering Research Center for Molecular Medicine, School of Basic Medical Science, Guizhou Medical University, Guiyang 550025, China

^4^ School of Pediatrics, Guizhou Medical University, Guiyang 550025, China

Corresponding Author

*Li Lei−Department of Chemistry, Engineering Research Center for Molecular Medicine, School of Basic Medical Science, Guizhou Medical University, Guiyang 550025, China

Phone: +86 15900917585; Email: [1002087851@qq.com;](mailto:1002087851@qq.com;)

^†^Song Xiang and Xincheng Long contributed equally to this paper

**Table of Contents**

**Fig. S1, S2…………………………………………………………………**3

**Fig. S3, S4…………………………………………………………………**4

**Fig. S5, S6…………………………………………………………………**5

**Fig. S7, S8…………………………………………………………………**6

**Fig. S9, S10………………………………………………………………..**7

**Fig. S11 and Table S1…………………………………………………….**8


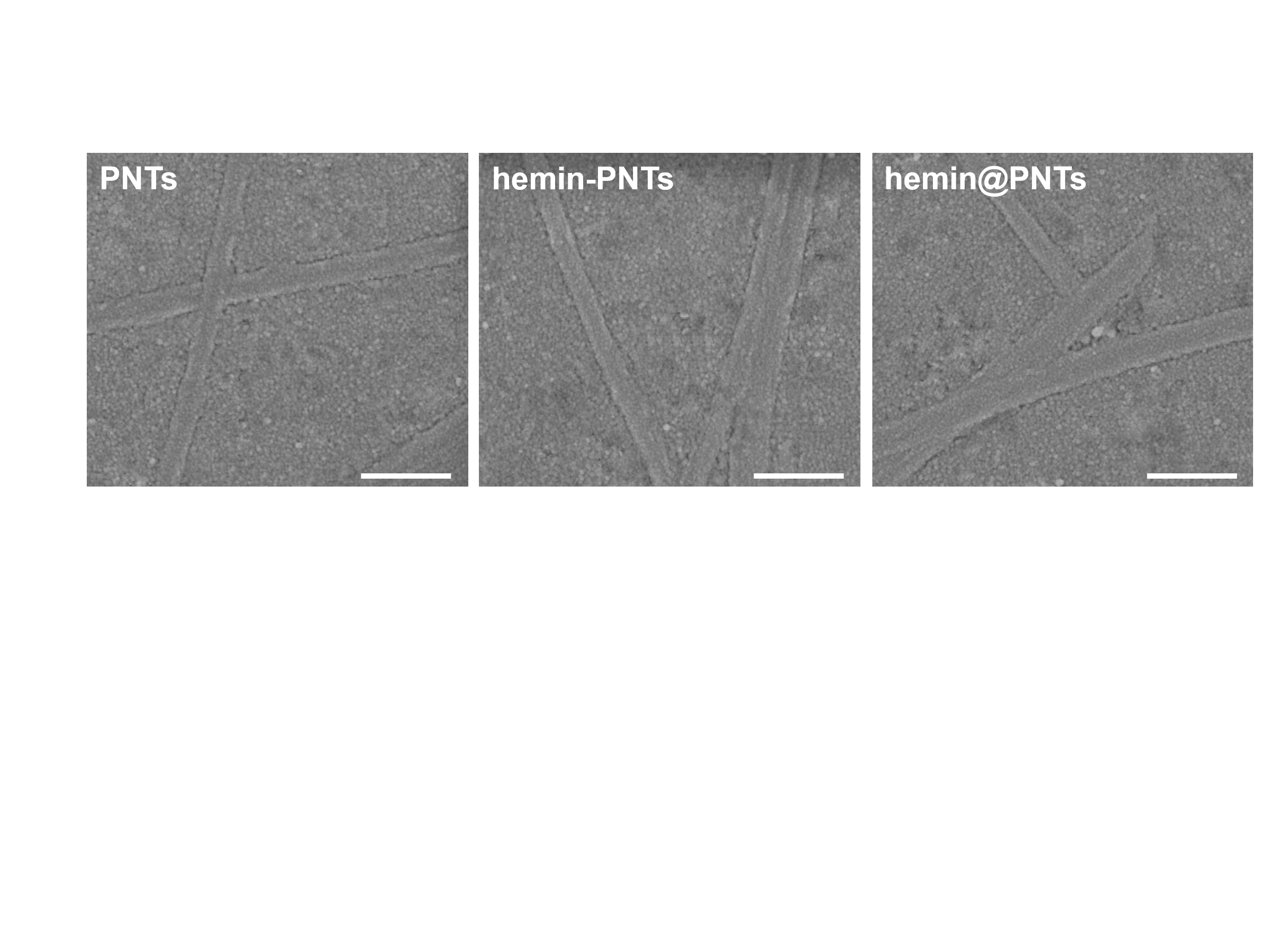


**Fig. S1** SEM images of PNTs, hemin-PNTs and hemin@PNTs, scale bars = 250 nm.


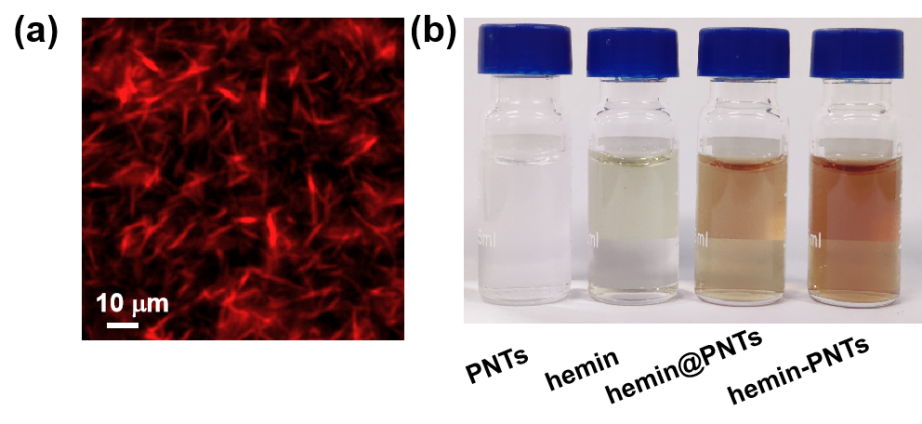


**Fig. S2** (a) Fluorescence micrograph of 1 mM PNTs incubated with 10 µM Nile red. (b) Photographs of PNTs, hemin, hemin-PNTs and hemin@PNTs.


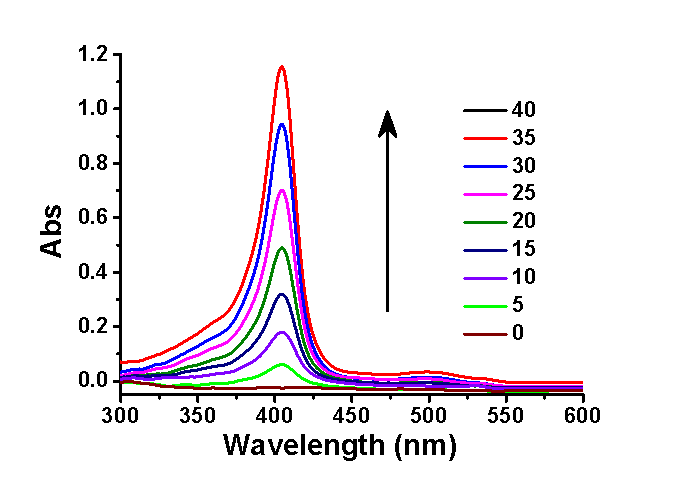


**Fig. S****3** UV–vis absorbance spectra of hemin (0-40 µg/mL).


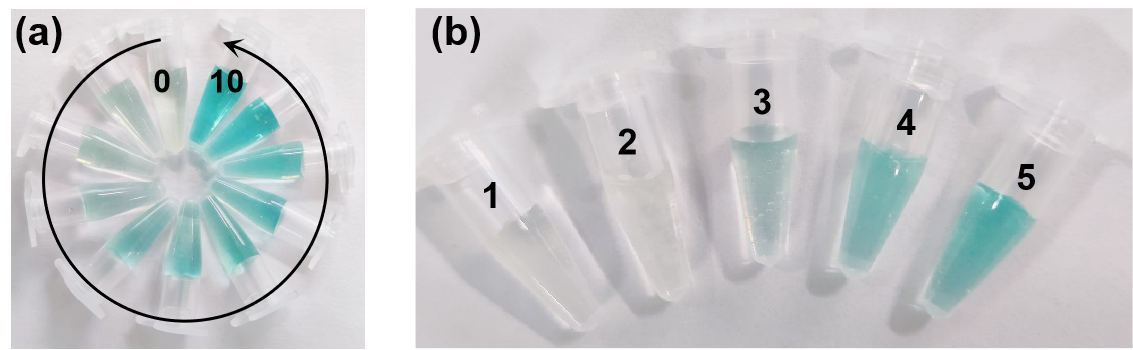


**Fig. S4** (a) The color change in the system of hemin-PNTs + TMB + H_2_O_2_ with the reaction time. (b) The optical photographs of the TMB–H_2_O_2_ mixed solution in the absence of a catalyst (1), and in the presence of PNTs (2), hemin (3), hemin@PNTs (4) and hemin-PNTs (5), respectively.


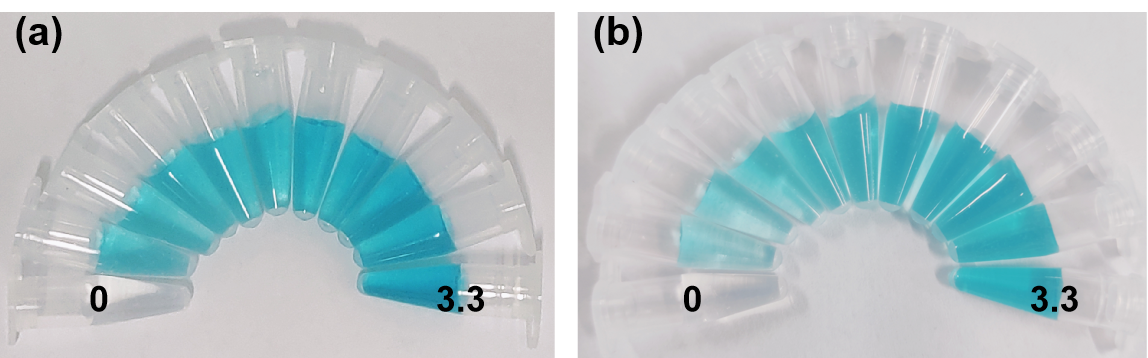


**Fig. S5** The optical photographs of the TMB–H_2_O_2_ mixed solution in the presence of increased concentration of hemin-PNTs (a) and hemin@PNTs (b).


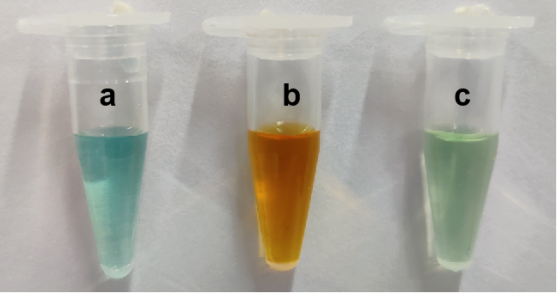


**Fig. S6** Hemin-PNTs-catalyzed oxidation of various substrates showing changes in color: (a) TMB, (b) OPD, (c) ABTS.


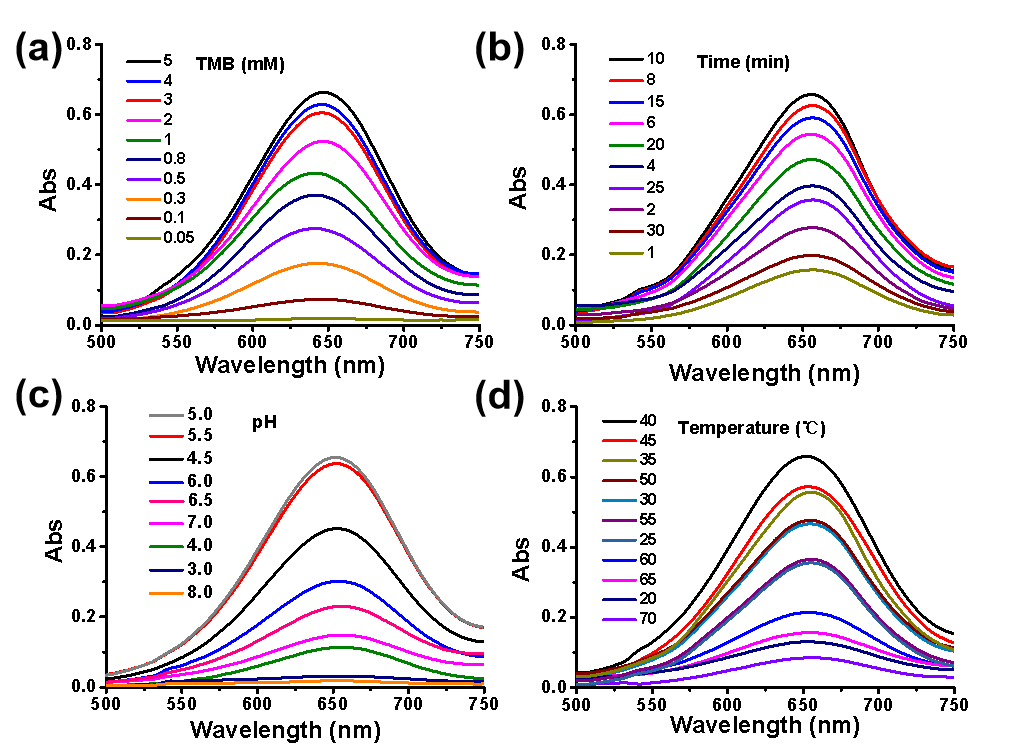


**Fig. S7** Effect of reaction TMB (a), time (b), pH(c), and temperature (d) on the peroxidase-like activity of hemin-PNTs for the TMB oxidation. The experiment was carried out using 3.3 μg/mL hemin-PNTs in a reaction volume of 1.0 mL, in tris-HCl buffer (1 mM, pH 5.0) with 3.0 mM TMB and 1 mM H_2_O_2_ for 10 min at 40 °C.


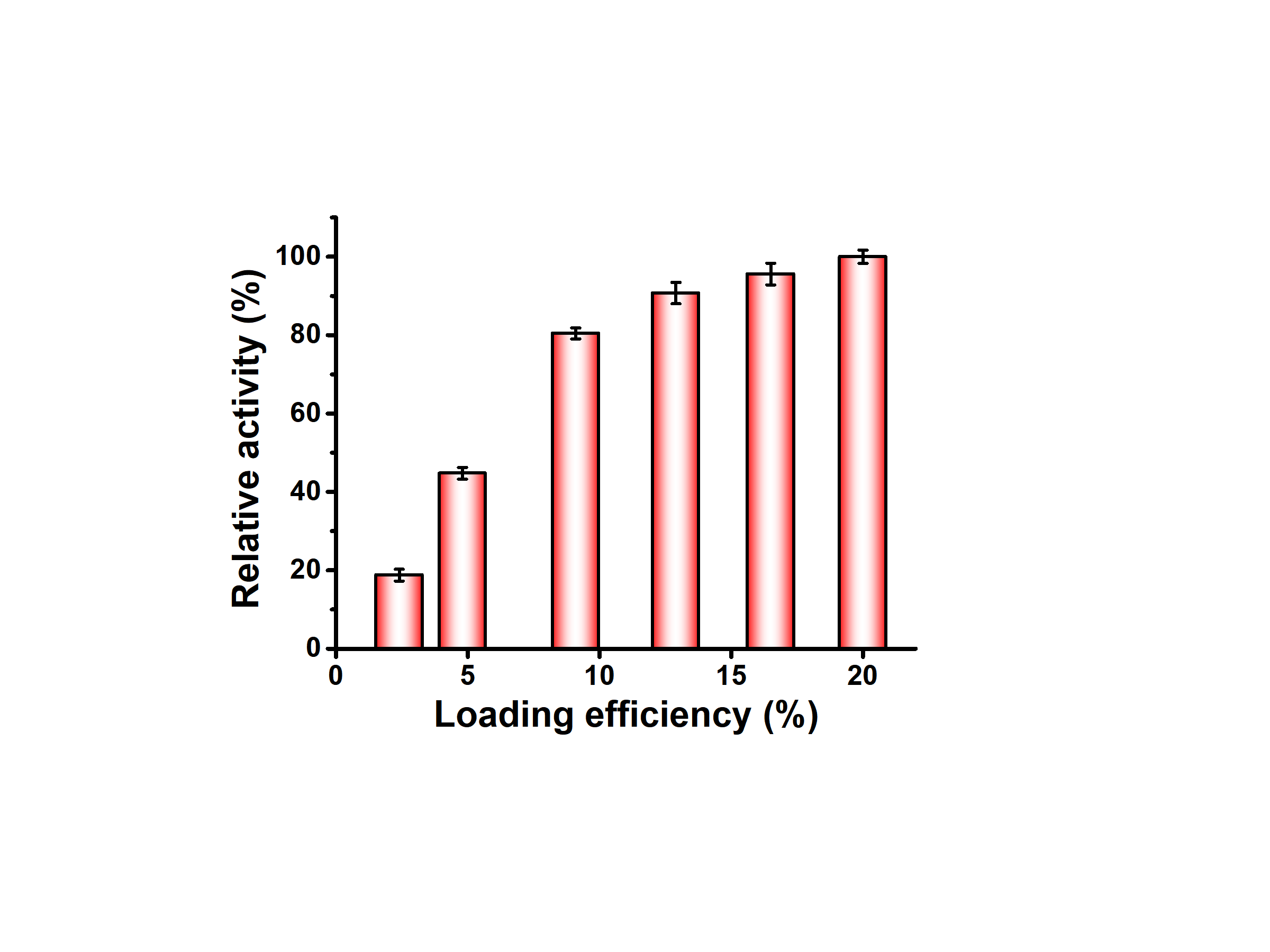


**Fig. S8** The catalytic activity of hemin-PNTs with different hemin loading efficiency.


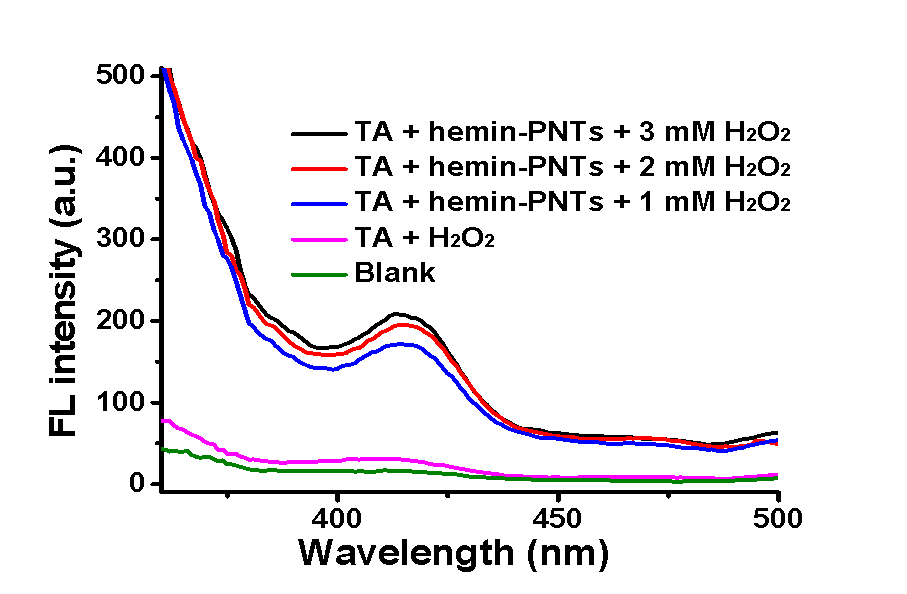


**Fig. S9** A Proof for the enhanced generation of **^.^**OH radicals by photolutuminesence spectral changes of terephthalic acid solution in the presence of H_2_O_2_ with different concentration.


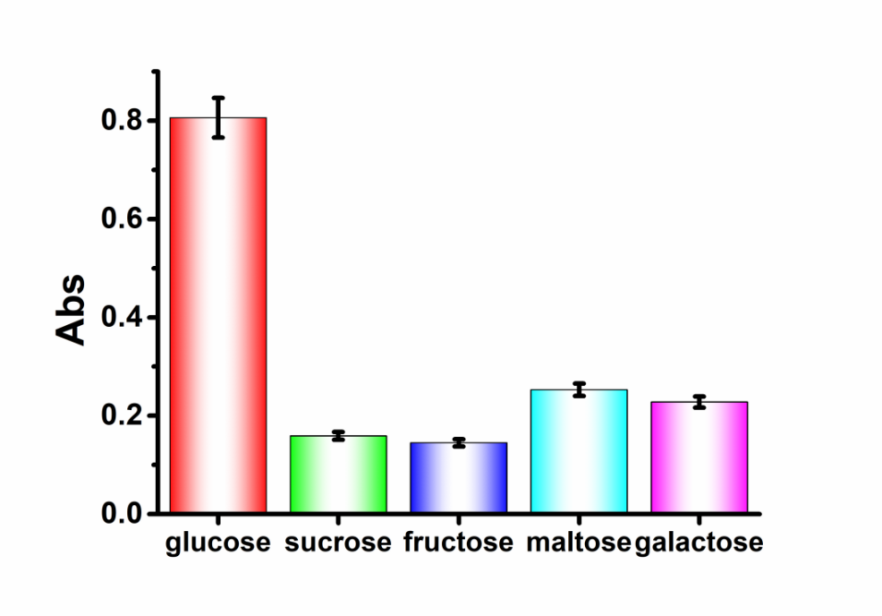


**Fig. S10** A Selectivity analysis glucose, detection by monitoring the absorbance at 652 nm. The analyte concentrations were as follows: 1 mM glucose, 10 mM the concentration of interferences ( sucrose, fructose, maltose, galactose).

**
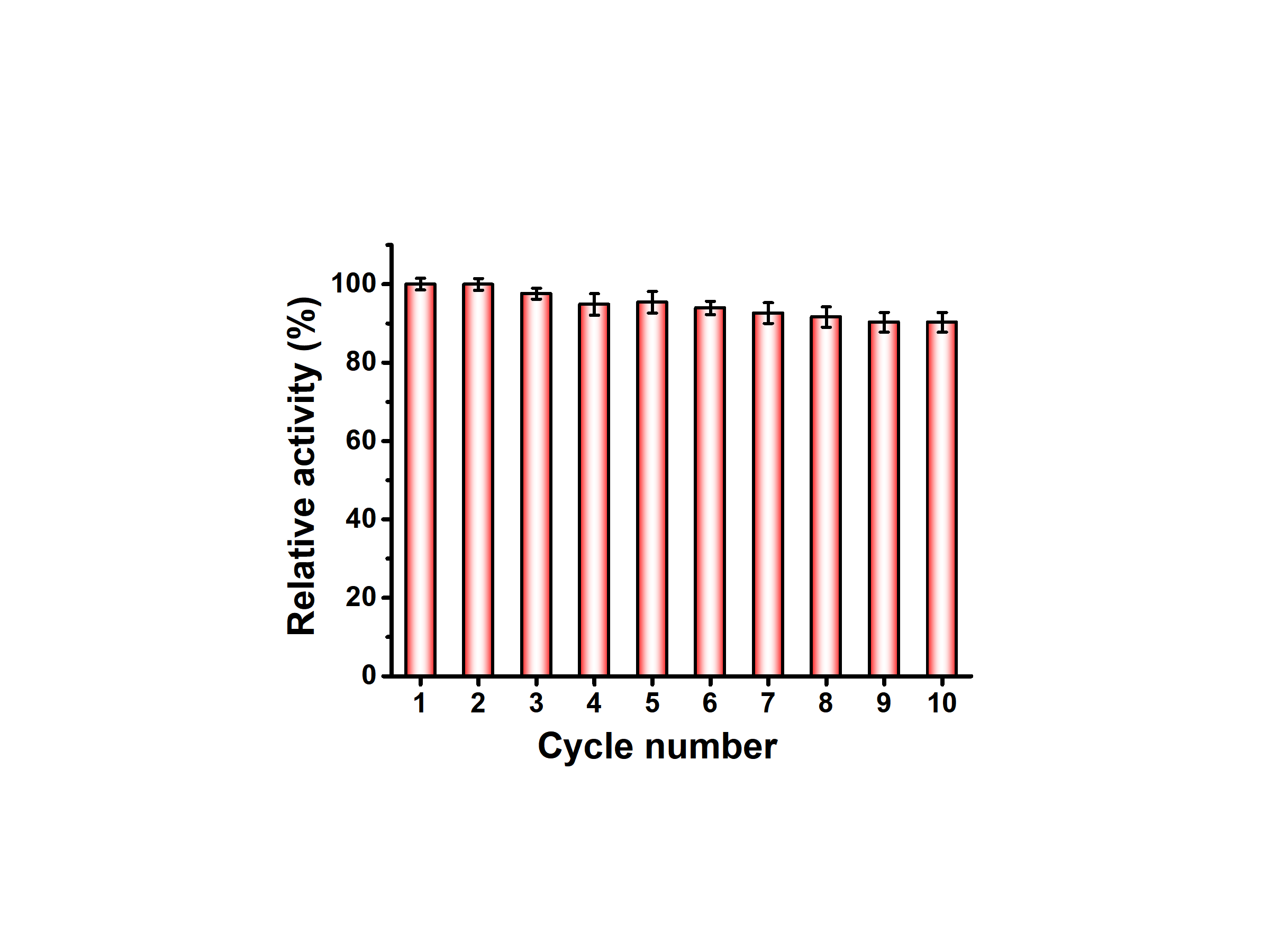
**

**Fig. S11** Recyclability of hemin-PNTs.

**Table S1** The loading efficiencies (LE %) calculated by formula of LE (%) = (weight

of loaded hemin)/(total weight of PNT) × 100%.

| Samples | Molar ratio | | LE (%) | |
| --- | --- | --- | --- | --- |
|  | Hemin | KL-7 or PNTs | Hemin in hemin-PNTs | Hemin in hemin@PNTs |
| 1 | 1 | 3 | 2.4 | 2.3 |
| 2 | 1 | 4 | 4.8 | 4.1 |
| 3 | 1 | 5 | 9.1 | 8.4 |
| 4 | 1 | 8 | 12.9 | 10.8 |
| 5 | 1 | 15 | 16.5 | 12.7 |
| 6 | 1 | 30 | 20.0 | 13.6 |
